# IMU-Based Energy Expenditure Estimation for Various Walking Conditions Using a Hybrid CNN–LSTM Model

**DOI:** 10.3390/s24020414

**Published:** 2024-01-10

**Authors:** Chang June Lee, Jung Keun Lee

**Affiliations:** 1 Department of Integrated Systems Engineering, Hankyong National University, Anseong 17579, Republic of Korea; cjlee@hknu.ac.kr; 2 School of ICT, Robotics & Mechanical Engineering, Hankyong National University, Anseong 17579, Republic of Korea

**Keywords:** energy expenditure estimation, inertial measurement unit, convolutional neural network, long short-term memory, walking and running

## Abstract

In ubiquitous healthcare systems, energy expenditure estimation based on wearable sensors such as inertial measurement units (IMUs) is important for monitoring the intensity of physical activity. Although several studies have reported data-driven methods to estimate energy expenditure during activities of daily living using wearable sensor signals, few have evaluated the performance while walking at various speeds and inclines. In this study, we present a hybrid model comprising a convolutional neural network (CNN) and long short-term memory (LSTM) to estimate the steady-state energy expenditure under various walking conditions based solely on IMU data. To implement and evaluate the model, we performed level/inclined walking and level running experiments on a treadmill. With regard to the model inputs, the performance of the proposed model based on fixed-size sequential data was compared with that of a method based on stride-segmented data under different conditions in terms of the sensor location, input sequence format, and neural network model. Based on the experimental results, the following conclusions were drawn: (i) the CNN–LSTM model using a two-second sequence from the IMU attached to the lower body yielded optimal performance, and (ii) although the stride-segmented data-based method showed superior performance, the performance difference between the two methods was not significant; therefore, the proposed model based on fixed-size sequential data may be considered more practical as it does not require heel-strike detection.

## 1. Introduction

In recent years, the importance of health management has been highlighted, and ubiquitous healthcare systems that continuously monitor health status and remotely diagnose diseases using wearable devices such as smartwatches have received considerable attention [1,2,3]. To this end, technologies for monitoring the intensity of physical activity are widely used to record the amount of exercise or to evaluate motor performance [4,5,6]. To quantify the intensity of physical activity, it is important to estimate energy expenditure (EE), which is the amount of energy or calories burned per time period (for example, watts or kcal/min). EE is also used to evaluate exoskeleton assistance and optimize the control strategies for personalized exoskeletons [7,8,9,10].

The EE can be calculated based on the volume of oxygen uptake (VO_2_) and carbon dioxide output (VCO_2_) measured using a metabolic gas analysis system [11]. This system is regarded as the gold standard because of its high accuracy. However, it requires expensive equipment and wearing a mask to measure the volume of oxygen and carbon dioxide; therefore, its use during activities of daily life is limited. To overcome these limitations, studies have been conducted to estimate EE using wearable sensors, such as inertial measurement units (IMU) or electromyography (EMG) sensors [12,13,14,15,16,17,18]. Such sensors are small, lightweight, and relatively low-cost; therefore, they can be easily applied to a variety of wearable devices and used in daily life.

An IMU comprising an accelerometer and a gyroscope can estimate the kinematics [19,20,21] and kinetics [22,23,24] of human motion, which have been found to be correlated with the EE [25,26,27]. Accordingly, several studies have developed algorithms to estimate EE based on IMU signals using linear regression or machine learning techniques [12,13,14,15,16,17,18]. Most algorithms extract the sum or features from IMU signals during a certain period for use as inputs to the model. For example, the ActiGraph (Pensacola, FL, USA) accelerometer provides a count that is the sum of post-filtered sensor signals within given time intervals, and several studies have used this count as the input or extracted features from the count to estimate the EE [12,13,14]. Ellis et al. [15] and Montoye et al. [16] used a variety of features, such as the mean, standard deviation, and maximum and minimum values extracted from the time window of raw accelerometer signals, as input to data-driven models, including linear regression, random forest, and neural network models. Zhu et al. [17] developed a convolutional neural network (CNN) model that automatically extracted features for EE estimation from fixed-size sequences of accelerometer signals and heart rate data. Paraschiakos et al. [18] developed a recurrent neural network (RNN) that estimates EE from the standard deviation of accelerometer data and trained and tested the model on elderly people.

The results of the above studies show that EE during physical activities in daily life can be reliably estimated using IMU-based models. However, most studies aim to estimate EE as a means of evaluating the intensity of physical activity, and this does not require a high level of accuracy for each detailed activity condition. However, in evaluating exoskeleton assistance and optimizing the assistance patterns of personalized exoskeletons, it is necessary to rapidly estimate EE with high accuracy. Accordingly, recent research has been conducted to estimate EE during activities under various conditions, including different speeds, inclines, and exoskeleton assistance [28,29,30,31,32].

Ingraham et al. [29] presented a linear regression model to estimate EE during activities including level, inclined, and backward walking based on physiological signals such as the heart rate, skin temperature, electrodermal activity, acceleration, EMG, and respirometer data. Lopes et al. [30] proposed CNN and long short-term memory (LSTM) models that estimate EE for walking with and without exoskeleton assistance based on IMU, EMG, and heart rate sensor data. Slade et al. [31] proposed a method using EMG signals and vertical ground reaction force to estimate EE during inclined, loaded, and assisted walking with different assistance patterns. In a follow-up study [32], a low-cost IMU-based wearable system was developed to estimate EE during steady-state and time-varying activities, including level walking/running, stair climbing, and biking. Although these studies reported high performance for various walking conditions based on wearable physiological signals, no studies have reliably estimated the EE while walking at various inclines with a simple sensor configuration, for example, a single IMU. 

This study presents a neural network model that estimates the steady-state EE during level/inclined walking and level running based on IMU signals. The proposed model employs a hybrid architecture of CNN and LSTM to estimate EE from sequential IMU data of a fixed size. To collect data for the model implementation and evaluation, we performed walking and running experiments on a treadmill with seven healthy male subjects. The performance of the models was compared for each of the five sensor locations. We performed a leave-one-subject-out cross-validation to evaluate the performance of the proposed model on unexperienced new data.

## 2. Materials and Methods

### 2.1. Data Collection

In this study, we conducted walking and running experiments on a treadmill to collect data for the implementation and evaluation of the proposed model. Seven healthy male subjects (age: 24.4 ± 1.7 years, height: 1.73 ± 0.06 m, weight: 72.8 ± 6.3 kg) participated in the experiment. All subjects performed level walking at two speeds (4 and 6 KPH), level running at two speeds (7.5 and 9 KPH), inclined walking at two speeds (4 and 6 KPH), and two inclines (3 and 6%). The walking experiment lasted 10 min, and the running experiment lasted 8 min. In all experiments, the subjects were asked to stand still for the first and last minutes. The order of the experiments was random. To ensure stable respiration, the subjects were asked to rest for at least three minutes before each experiment. This study was approved by the Public Institutional Review Board of the Ministry of Health and Welfare of Korea (P01-202110-13-001).

**Figure 1:**
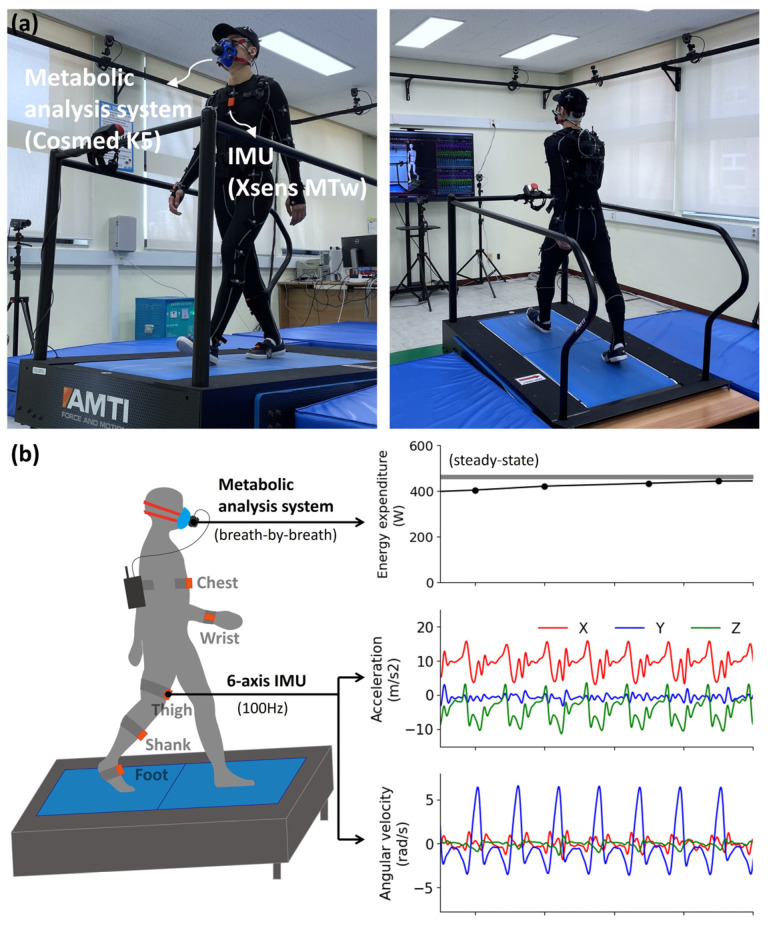
(**a**) Experimental setup and (**b**) depiction of data collection.

During the experiments, we collected data from a six-axis IMU module (MTw, Xsens Technologies B. V., Enschede, The Netherlands) comprising an accelerometer, gyroscope, and metabolic gas analysis system (K5, COSMED, Rome, Italy). Five IMU modules were attached to the chest, right wrist, thigh, shank, and foot of the right leg using a Velcro strap band. Six-axis IMU data were collected at a sampling rate of 100 Hz and filtered through a fourth-order Butterworth low-pass filter with a cutoff frequency of 6 Hz. The oxygen intake and carbon dioxide output were measured breath by breath (mL/min) using a metabolic analysis system. The EE was calculated in watts using the Brockway equation [11] based on the oxygen intake and carbon dioxide output. The ground truth of the EE for each experimental condition was determined by averaging the EE results over the last four minutes of steady-state walking/running. Figure 1 shows the experimental setup and depiction of the data collection.

### 2.2. Model Architecture

This study implemented a data-driven model using a CNN and LSTM to estimate the EE from sequential IMU data. CNN is a type of neural network architecture commonly used in computer vision tasks or time series estimation [33,34]. CNNs typically comprise a convolution layer and a maximum or average pooling layer. They each have a fixed-size kernel that is used to identify meaningful features or patterns from images or sequential data. In sequential data processing, a one-dimensional (1D) CNN is applied that moves the kernel by a stride over time. Another neural network architecture useful for time series data is an RNN. A basic RNN has an internal memory that stores the past state information, which is combined with current information to output the current state. However, this network has a long-term dependency problem, and thus, modified RNNs have been developed to solve this problem. LSTM is one of the RNNs modified to capture long-term dependencies of sequential data [35,36]. This network has long- and short-term states, and three different gates for input processing including input, output, and forgetting gates. CNN and LSTM are both effective neural network models for time series data estimation problems; however, they differ in the processing of input data. To combine the advantages of both models, hybrid architectures of CNN and LSTM have been developed in recent research [37,38].

**Figure 2:**
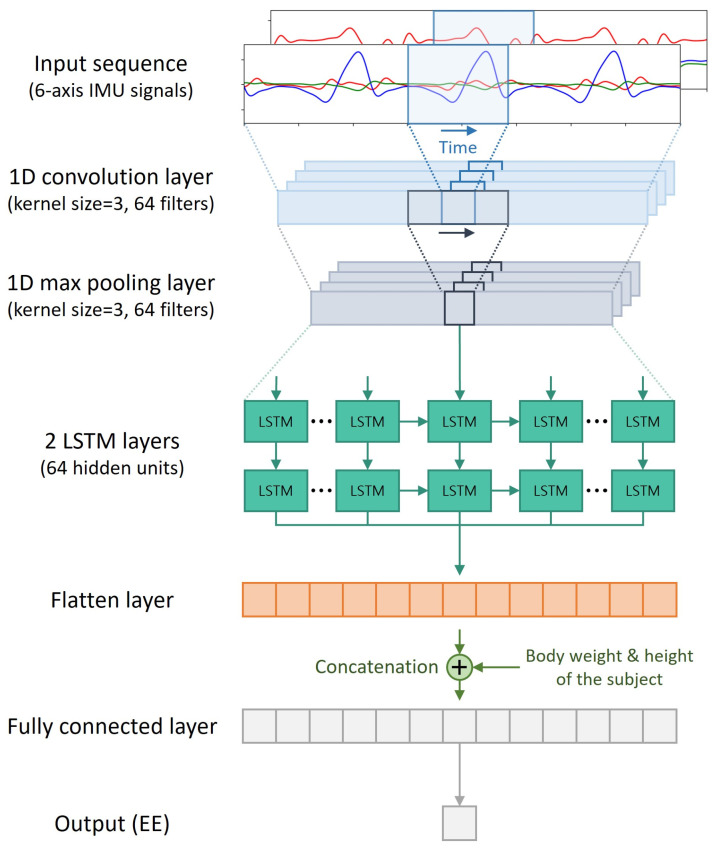
Architecture of the presented CNN–LSTM model.

This study adopted a CNN to extract features from a sequence of IMU signals and LSTM to estimate the EE based on sequential features from the CNN layer. First, the input layer feeds a fixed-size sequence from six-axis IMU signals comprising three-dimensional (3D) linear acceleration and 3D angular velocity signals. Then, a 1D convolution layer and a 1D maximum pooling layer move their kernels onto the sequential input data. Subsequently, the two LSTM layers pass the sequential features extracted from the CNN layers and flatten them to a 1D vector through a flattened layer. Finally, the flattened vector was concatenated with the body weight and height of the subject and passed through a fully connected layer to estimate the EE. The convolution and maximum pooling layers both have 64 filters with a kernel size of 3 and stride of 1, and the two LSTM layers both have 64 hidden units. Figure 2 shows the architecture of the CNN–LSTM model. 

To achieve optimal EE performance, it is important to select the appropriate sampling rate and time of the input sequence as well as the appropriate model architecture. For example, although longer sequence times and higher sampling rates provide more information, they do not necessarily guarantee high performance. We compared three sequence times (2, 4, and 6 s) and three sampling rates (60, 80, and 100 Hz) to determine the optimal input sequence format.

### 2.3. Model Implementation and Evaluation

The proposed CNN–LSTM model was implemented by updating the model parameters to minimize the mean squared error (MSE) between the predicted EE and ground truth of the EE from the metabolic analysis system. In the training process, adaptive moment estimation (commonly called Adam) was selected as the optimization technique to update the model parameters. To determine the optimal model parameters quickly, we applied one-cycle scheduling, a technique that adjusts the learning rate according to the epochs. We set the maximum learning rate to 0.001 and the number of epochs to 100. During model training, a dropout of 0.5 was applied between each LSTM layer and the fully connected layer, and L2 regularization was applied to prevent the model overfitting problem. In the training dataset, the input and target data were rescaled to ensure training stability. The IMU data were standardized by subtracting the mean and dividing by the standard deviation to reduce the effects of scale differences in between each axis signal. The ground truth of the EE and subject-specific data was normalized to a range between 0 and 1. The model was implemented using PyTorch 1.7.1, a Python-based deep-learning framework.

In this study, we performed leave-one-subject-out cross-validation to evaluate the performance of the proposed model using new data. The performance of the model was evaluated using the normalized root-mean-square error (NRMSE) of the body weight of the subject in W/kg, and the mean absolute percentage error (MAPE) in %. The NRMSE and MAPE were calculated from the results during steady-state walking and running, excluding the static and transition states.

The performance of the proposed model is compared and analyzed in two sections. In the first section, we compare the estimation performance depending on the sequence time and sampling rate of the input sequence of the proposed model. By comparing the performance at three sequence times and three sampling rates, we determined the optimal input-sequence format. We also compared the performance of the hybrid CNN–LSTM with those of the CNN and LSTM models to verify the suitability of adopting CNN–LSTM. Both models comprised three hidden layers (1D convolution layer and 1D maximum pooling layer for the CNN model and LSTM layer for the LSTM model), a flattened layer, and a fully connected layer. In the second section, the performance of the proposed model is compared with that of a method that uses different types of input data for EE estimation [31,32]. This method uses sensor signals during each gait cycle as inputs to the model through the segmentation of the signals for each stride. Based on this method, we implemented a multilayer perceptron (MLP) model comprising an input layer feeding stride-segmented IMU signals (size 30) and subject-specific data, a flattened layer, three hidden layers with 400 neurons, rectified linear unit (commonly called, ReLU) functions, and a fully connected layer. To implement the MLP model, the six-axis IMU signals were segmented by each stride based on the shank-attached gyroscope signal by applying the gait detection technique from [39]. Additionally, we also compared the performance of the models on the following three training and testing datasets: (Dataset 1) level and inclined walking data, (Dataset 2) level walking and running data, and (Dataset 3) all data. In both sections, performance comparisons were conducted according to the sensor attachment locations.

## 3. Results

### 3.1. Comparison by the Input Sequence and Neural Network Model

In this section, we compare the estimation performance of the EE for different sequence times and sampling rates of the input sequence and then determine the optimal input sequence format. Nine cases were compared, with sequence times of 2, 4, and 6 s and sampling rates of 60, 80, and 100 Hz. 

**Figure 3:**
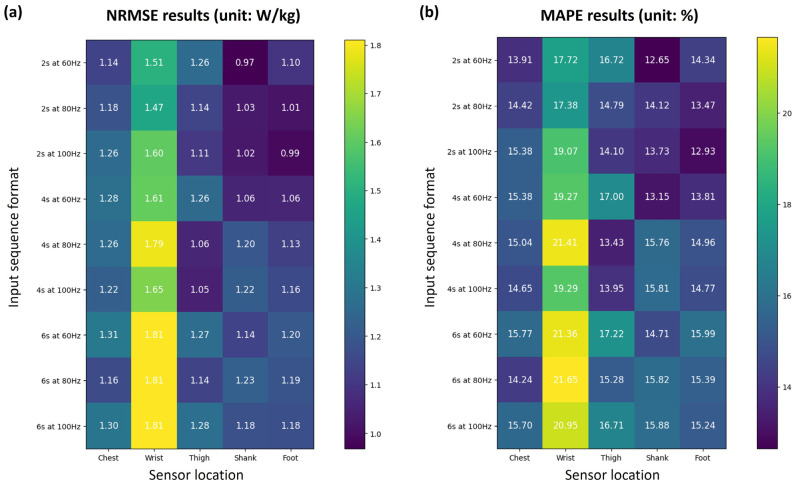
Results of (**a**) NRMSE in W/kg and (**b**) MAPE in % depending on the input sequence format and sensor location.

Figure 3 shows the NRMSE and MAPE results of the estimated EE for nine input sequence types and five sensor attachment locations. In most cases, the estimation performance for each sensor location was better for lower-body segments than for the wrist or chest. The wrist showed the highest errors (NRMSE > 1.45 W/kg and MAPE > 17.35%) in all cases, and the chest showed an average NRMSE of 1.24 W/kg and an average MAPE of 14.94%. Among the three lower-body segments, the average performance (NRMSE and MAPE) in all cases was high in the following order: foot (1.11 W/kg and 14.54%), shank (1.11 W/kg and 14.63%), and thigh (1.17 W/kg and 15.47%). 

The results of the comparison by sequence time showed that performance improved in short sequences compared to long sequences, especially for the foot and shank. For example, the NRMSE of the foot-attached model was 1.18 W/kg for a 6 s sequence at 100 Hz, while it was 0.99 W/kg for a 2 s sequence at 100 Hz, which is an improvement of approximately 15.8%. For the thigh-attached models, 4 s sequences showed higher performance than the others. However, no consistent trends or patterns were identified in the results compared to the sampling rate. For example, for 2 s sequences, the shank and foot attachment models showed the highest performance at 60 Hz and 100 Hz, respectively.

Among all input sequence formats and sensor locations, the shank-attached model using a 2 s sequence at 60 Hz showed the highest performance (0.97 W/kg of NRMSE and 12.65% of MAPE). In terms of the average performance of the three lower-body segments, the 2 s sequence at 100 Hz had the highest performance. Accordingly, we selected the 2 s sequence at 100 Hz as the optimal input sequence format for EE estimation.

We also compared the performance of three types of neural network models: CNN, LSTM, and hybrid CNN–LSTM models. Table 1 lists the NRMSE and MAPE results of the three neural network models that use a 2 s sequence of IMU signals at 100 Hz as the model input. The hybrid CNN–LSTM model exhibited the highest performance among the three models in terms of the average NRMSE and MAPE for the five sensor locations. For the wrist and thigh attachments, although the CNN showed a lower NRMSE than the hybrid model, the hybrid model showed higher performance in terms of MAPE. The difference between these two results was due to the difference in the estimation error of the normalization method (normalized by body weight for the NRMSE and the truth value for the MAPE).

### 3.2. Comparison with the Method Based on Stride-Segmented Data

In this section, we compare the performance of the proposed model with that of a method that uses the IMU signals segmented by each stride. Table 2 lists the NRMSE and MAPE results of the MLP model using stride-segmented data and the presented CNN–LSTM model on the three training and testing datasets. For the training model on Dataset 3, the testing results of Datasets 1–3 were compared.

First, in the training/testing results of Dataset 1, both methods showed relatively high performance in the lower-body segments, especially the shank and foot, compared to the chest and wrist as sensor locations. This tendency is observed also in the results in the previous section. The shank attachment produced the highest performance for both MLP (0.86 W/kg and 12.28%) and CNN–LSTM (0.91 W/kg and 12.98%). Second, in the training/testing results of Dataset 2, the MLP showed the best performance for the shank attachment (0.93 W/kg and 10.67%), similar to the results of Dataset 1. In contrast, the CNN–LSTM model showed higher performance for the wrist (0.94 W/kg and 10.56%) compared to other lower-body segments (≥0.98 W/kg and 13.04%). Third, in the training results of Dataset 3, the sensor locations that exhibited the high performance for the three testing datasets and both models were the shank or foot. Overall, the performance of the CNN–LSTM model was inferior to that of the MLP for most sensor locations except for the chest. Nevertheless, for the lower-body segments, the performance difference between the two models was insignificant for some datasets. For example, in the foot attachment, the difference in NRMSE between the two models was approximately 0.2 W/kg for Dataset 2, while it was less than 0.04 W/kg for Datasets 1 and 3, which can be considered equivalent performance.

**Figure 4:**
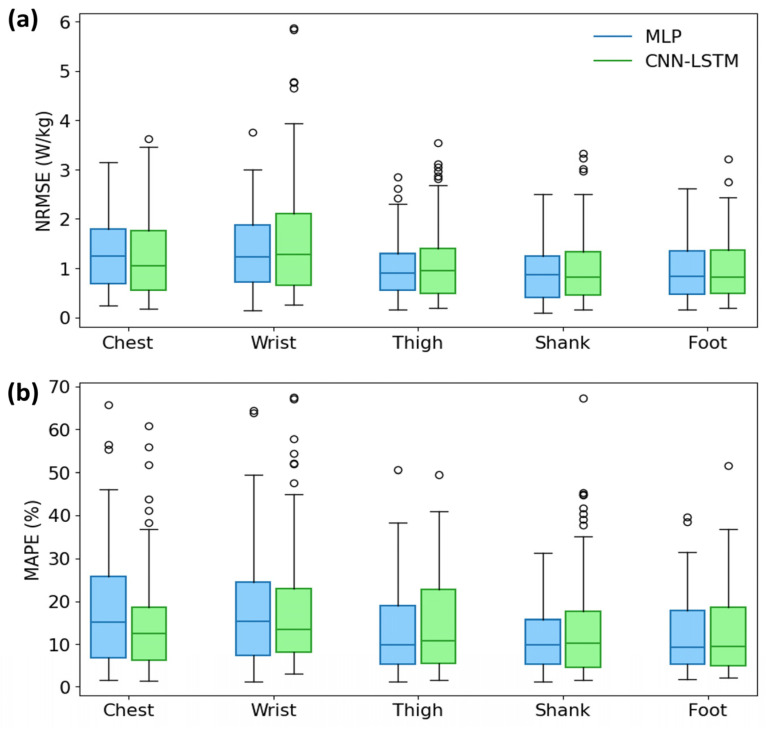
Boxplot results in comparison of (**a**) NRMSE and (**b**) MAPE between the MLP using stride-segmented data and the presented CNN–LSTM models.

Figure 4 shows the boxplot results of the comparison of the NRMSE and MAPE between both models for Dataset 3. The boxplot results show that the estimation error of the proposed model is higher and more widely distributed than that of the MLP. However, the difference in performance was not significant between the shank and foot attachments.

**Figure 5:**
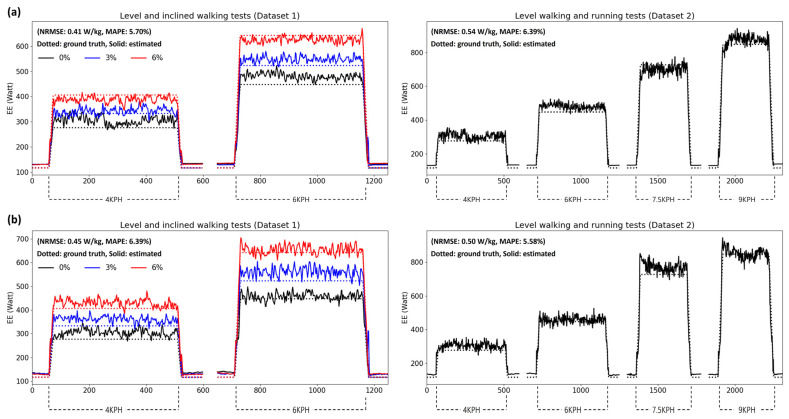
Estimation results of subject data showing good performance from the presented model based on (**a**) foot- and (**b**) shank-attached IMUs.

**Figure 6:**
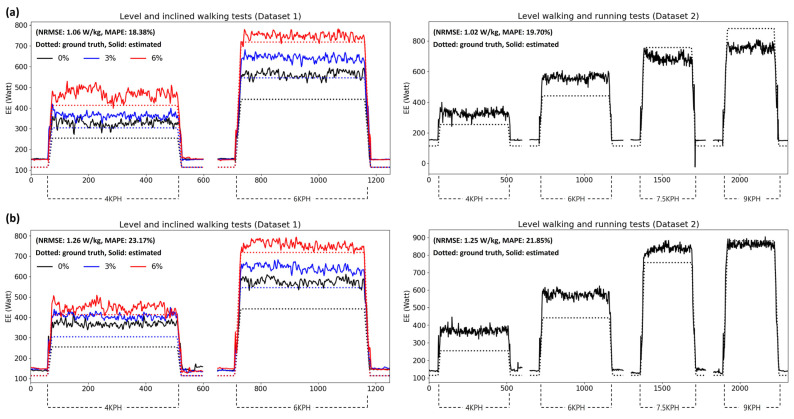
Estimation results of subject data showing bad performance from the presented model based on (**a**) foot- and (**b**) shank-attached IMUs.

Figure 5 and Figure 6 show the estimation results for the subject data showing good (MAPE < 7%) and bad (MAPE > 18%) performances, respectively, from the presented model. Both figures show the estimation results of Datasets 1 and 2 based on foot- and shank-attached IMUs. In Figure 5, the results of both models converge to the ground truth of the steady-state EE for each walking speed and inclination, although they are noisy. However, as shown in Figure 6, the models overestimated the EE while walking higher than the ground truth, resulting in biased errors. Nevertheless, the models showed results that partially reflected changes in the EE depending on the walking conditions.

## 4. Discussion

In this study, the performance of the proposed CNN–LSTM model was compared by sensor location, input sequence format, and neural network model, and with that of the MLP model using stride-segmented data as input. 

Regarding the sensor attachment location, the lower-body segments, especially the shank and foot, mostly showed higher performance than the chest and wrist. The movements of the shank and foot, which are the lower-body segments close to the ground, may be significantly affected by the inclination of the ground and walking or running speeds. Therefore, the movement patterns of the two segments are expected to be clear for each inclination and speed. As an exception, the wrist attachment produced better performance than the lower-body attachments for the CNN–LSTM model on the level walking and running data. Upper-body segments, including the chest and wrist, may also have different patterns of motion depending on the walking or running speed. However, since the results showed that both segment-attached models exhibited poor performance for datasets including inclined walking data (Datasets 1 and 3), it is assumed that their patterns of motion are not clear for each incline.

As the input sequence format, a 2 s sequence showed the best performance among the three sequence times (2, 4, and 6 s) for the shank- and foot-attached IMUs. The foot-attached model showed the highest performance at a sampling rate of 100 Hz, whereas the shank-attached model showed an insignificant difference in performance among the three sampling rates (60, 80, and 100 Hz) for the 2 s sequence. These results demonstrate that the proposed model can use IMU data with short sequence times and low sampling rates for EE estimation, although long sequences with high sampling rates provide more information. Additionally, among the three types of neural network models, the hybrid CNN–LSTM model exhibited the best performance, indicating that it is reasonable to adopt the architecture of the hybrid model for EE estimation problems. 

By comparing the presented model with a method based on stride-segmented data [31,32], it was confirmed that data during each gait cycle can achieve superior performance to sequential data of fixed size as a model input for various walking conditions. However, it is necessary to detect heel strikes to segment the data for each stride. A variety of IMU-based gait detection techniques have been developed [39,40,41,42]; however, they still have inaccuracies that can lead to performance deterioration in EE estimation. Particularly, walking and running conditions including more diverse inclines and speeds than the conditions of our experiments may make it challenging to accurately detect heel strikes. By contrast, the proposed model uses fixed-size sequences that require no additional processing for input formatting. Considering that the performance difference between the two methods is not significant (NRMSE from the foot attachment: 0.95 W/kg for MLP, 0.99 W/kg for CNN–LSTM), the method based on the fixed-size sequences may be considered more practical.

As described in the Introduction, several recent studies have been conducted to estimate EE under various walking conditions [29,30,31,32]. Ingraham et al. [29] presented a linear regression model to estimate EE for six activities including level, incline, and backward walking, learning, cycling, and stair climbing at various speeds or intensities (a total of 19 conditions). The authors compared various physiological signals and concluded that high performance can be achieved by combining a small number of sensors. Their combined sensor signals that showed optimal performance include respirometer data and EMG signals from eight bilateral lower-limb muscles. Loopes et al. [30] developed CNN and LSTM models to estimate EE during slow walking with and without assistance from an ankle exoskeleton at three speeds: 0.22, 0.33, and 0.44 m/s. They used data from IMUs on the sternum, pelvis, and lower limbs, EMG sensors on the four muscles of both legs, and a heart rate sensor. Although the above two studies reported reliable EE performance for a variety of activity conditions (NRMSE of 1.03–1.24 W/kg for [29] and 0.36 W/kg for [30]), their sensor configurations can be burdensome to users. Slade et al. [31] developed an MLP model based on EMG signals and vertical ground reaction force and evaluated the EE performance of the model on two datasets: (i) unilateral ankle-assisted walking with nine different assistance strategies and (ii) unassisted walking under three inclines (0, 5, and 10%) and four loading conditions (0, 10, 20, and 30% of their bodyweight). The authors reported NRMSE of 0.40 and 0.83 W/kg for novel subject data in the two datasets, respectively, showing that high EE performance can be achieved for various walking conditions, including additional external loads. However, this study also uses a sensor configuration including EMGs attached to the lower-limb segments and a force sensor that is difficult for users to use in everyday life. The follow-up study [32] developed a low-cost IMU-based system that is simpler and easier to don and doff than the system of their previous study [31]. The performance of the developed system was evaluated for level walking and running under steady-state and time-varying conditions, stair climbing, and biking, and the system produced a MAPE of 13%. This system achieves reliable EE performance using a simple sensor configuration but requires a procedure to segment sensor data by each stride, as discussed above. 

In contrast, the proposed method uses a simple sensor configuration and a simple input format, which is more effective than previous studies in terms of user convenience or signal processing. In particular, because the proposed method uses only a single-IMU dataset, it is expected that the method will be easier to implement in a real-time wearable system for continuous monitoring of EE compared to multiple-sensor-based methods. However, it is difficult for IMU-based systems to reflect the effects of activity conditions not addressed in this study, such as additional external loads, in EE estimation. Therefore, multiple-sensor configuration is essential to take into account more diverse activity conditions; for example, one solution to consider external loading conditions in EE estimation is to incorporate an additional insole pressure sensor that provides vertical ground reaction force. Nevertheless, this study confirmed that a data-driven method based on 2 s sequential data from a single IMU can reliably estimate the steady-state EE during walking at various inclines and speeds; this could allow researchers to have a wider choice of EE methods depending on the activities to be analyzed.

This is a preliminary study to verify the effectiveness of the proposed architecture in estimating EE during steady-state level and inclined walking on a treadmill. For this rea-son, experimental validation of this study only focused on EE estimation during walking on a treadmill. However, for practical applicability of the proposed architecture, the estimation performance for real-world conditions such as ground walking should be demonstrated. If ground walking is performed close to steady-state conditions, it is expected that the proposed architecture may achieve estimation performance similar to that in the results of this study. However, walking speed in real-world ground walking can frequently change, which will lead to inaccurate estimates. Therefore, similar to [32], the architecture must be developed to respond not only steady-state conditions but also time-varying conditions including inconsistent walking speed and inclination as well as uneven grounds. 

## 5. Conclusions

In this study, we presented a hybrid CNN–LSTM model to estimate the EE for various walking conditions. To implement and evaluate the model, we performed level/inclined walking and level running experiments on a treadmill. The performance of the proposed model was compared in terms of sensor location, input sequence format, and neural network model, and with that of a method using stride-segmented data as input. First, we found that the CNN–LSTM model using the 2 s sequence from the IMU attached to the lower body, that is, the shank and foot, had optimal performance. Second, the stride-segmented data-based method exhibited superior performance compared to the proposed model. However, the performance difference between the two methods was not significant; therefore, the proposed model using fixed-size sequences may be considered more practical as it does not require heel-strike detection. 

One limitation of this study is that the model was implemented and evaluated on only seven male subjects. Future work will aim to collect data from a wider range of subjects for the generalization of the model and to improve the performance of the model by optimizing the model architectures and hyperparameters. In future work, we will also collect data from the insole pressure sensor in addition to the IMUs to implement a model for EE estimation considering external loading conditions.

## Figures and Tables

**Table 1:** Results of NRMSE in W/kg (with MAPE in %) depending on the neural network type.

Neural Network Type	Chest	Wrist	Thigh	Shank	Foot
^a^ CNN	1.45 (16.86)	1.44 (16.26)	1.09 (15.04)	1.08 (14.62)	1.19 (15.59)
^a^ LSTM	1.32 (16.39)	1.59 (18.83)	1.62 (21.56)	1.40 (19.48)	1.31 (16.68)
CNN–LSTM	1.26 (15.38)	1.60 (19.07)	1.11 (14.10)	1.02 (13.73)	0.99 (12.93)

^a^ Both models comprised three hidden layers (1D convolution layer and 1D maximum pooling layer for the CNN model and LSTM layer for the LSTM model), a flattened layer, and a fully connected layer.

**Table 2:** Results of NRMSE in W/kg (with MAPE in %) from the MLP and the CNN–LSTM models on three training and testing datasets.

Training	Testing	Method	Sensor Locations				
			Chest	Wrist	Thigh	Shank	Foot
^a^ Dataset 1		MLP ^b^	1.27 (18.47)	1.57 (22.34)	1.07 (16.46)	0.86 (12.28)	1.02 (14.59)
		CNN–LSTM ^b^	1.11 (14.98)	1.71 (23.42)	1.18 (17.42)	0.91 (12.98)	0.90 (13.55)
^a^ Dataset 2		MLP	1.20 (15.98)	1.11 (13.79)	1.14 (14.36)	0.93 (10.67)	1.07 (12.82)
		CNN–LSTM	1.13 (14.40)	0.94 (10.56)	0.98 (13.04)	1.04 (13.59)	1.13 (14.80)
^a^ Dataset 3	Dataset 1	MLP	1.39 (20.72)	1.36 (19.62)	1.02 (15.54)	0.83 (11.81)	0.93 (13.34)
		CNN–LSTM	1.11 (15.63)	1.63 (21.23)	1.06 (15.19)	1.05 (15.69)	0.92 (13.86)
	Dataset 2	MLP	1.14 (15.93)	1.38 (18.05)	1.04 (14.12)	0.99 (11.93)	0.91 (11.25)
		CNN–LSTM	1.41 (16.64)	1.55 (18.83)	1.17 (14.93)	1.03 (13.86)	1.11 (14.06)
	Dataset 3	MLP	1.28 (17.49)	1.35 (17.51)	1.01 (13.71)	0.90 (11.17)	0.95 (12.18)
		CNN–LSTM	1.26 (15.38)	1.60 (19.07)	1.11 (14.10)	1.02 (13.73)	0.99 (12.93)

^a^ Dataset 1: level/inclined walking tests; Dataset 2: level walking/running tests; Dataset 3: all tests. ^b^ MLP: MLP model using stride-segmented data; CNN–LSTM: hybrid CNN–LSTM model using sequential data.

## Data Availability

The data presented in this study are available on request from the corresponding author. The data are not publicly available because it was collected and processed for this study.
